# Blocking leptin‐STAT3 axis‐induced fatty acid oxidation: A novel approach to activate CD8^+^ T effector cells in breast cancer

**DOI:** 10.1111/1759-7714.13691

**Published:** 2020-10-16

**Authors:** Ji Ruan

**Affiliations:** ^1^ Sun Yat‐sen University Cancer Center State Key Laboratory of Oncology in South China, Collaborative Innovation Center for Cancer Medicine Guangzhou China

Escaping immune surveillance and metabolic rewiring are considered to be the hallmark of cancer.^1^ They play a crucial role in the entire process of cancer development and progression to metastasis.^2^, ^3^ In recent years, restoring the desired immune response against cancer cells and targeting cancer‐specific metabolic reprogramming have been a focus for emerging therapeutic strategies.^4^ Growing evidence indicates that dietary abnormality‐associated obesity increases cancer risk and hinders anticancer treatment.^5^, ^6^ Additionally, the initiation and progression of obesity‐associated cancer are closely related to immune disorders.^5^ Indeed, the initiation of hepatocellular carcinoma, one of the obesity‐associated cancers, is largely due to the activation of oncogenic signal transducer and activator of transcription 3 (STAT3) caused by interleukin 6 and tumor necrosis factor (TNF)‐induced hepatic inflammation.^5^ Moreover, the association between obesity and programmed cell death protein 1 (PD‐1) mediated T cell dysfunction has been demonstrated in cancer.^7^, ^8^ However, how the excess of fatty acids associates with obesity to directly contribute to other obesity‐associated cancers, such as breast cancer, is still unknown. Furthermore, the mechanisms underlying T cell dysfunction in obesity remain to be explored.

As mentioned above, STAT3 can induce inflammation and lead to obesity‐associated HCC.^5^ Recent studies have also demonstrated that STAT3 is an important checkpoint for blocking antitumor immune responses in multiple immune cells.^9^ Silencing Stat3 in mouse CD8^+^ T cells can increase the infiltration and antitumor immunity of CD8^+^ T effector (T_EFF_) cells,^10^, ^11^ and the Th1 immune responses induced by STAT3 silencing in T cells are associated with increased expression of interferon‐gamma (IFN‐γ).^9^, ^11^ These evidences indicate that STAT3 is an important transcription factor that has a role in cancer‐promoting inflammation, but how STAT3 suppresses antitumor immunity to inhibit the expression of Th1 stimulating factors remains unclear.

Increasing evidence suggests that metabolic reprogramming can regulate T cell functions in cancer. For instance, the reduction of glycolysis in CD8^+^ T_EFF_ cells inhibits their antitumor activity.^12^ Extensive consumption of glucose by cancer cells reduces the glycolysis of CD8^+^ T cells, thereby inhibiting the production of IFN‐γ. IFN‐γ is a key regulator of CD8^+^ T_EFF_ cell proliferation and antitumor immunity. Chang *et al*.^12^ have demonstrated that anti‐PD‐1/programmed death‐ligand 1 (PD‐L1) antibodies can restore glucose consumption in the tumor microenvironment by reducing tumor volume, thereby enabling CD8^+^ T_EFF_ cell to resume glycolysis and IFN‐γ production. Furthermore, the enhancement of fatty acid oxidation (FAO) has been shown to improve the efficacy of adoptively transferred CD8^+^ T_EFF_ cells and PD‐1 blockade.^13^ The studies mentioned above have shown that cancer metabolism plays a very important role in regulating the function of CD8^+^ T cells. Therefore, it is necessary to further explore the metabolic mechanisms, especially the mechanisms associated with FAO, in regulating the functions of CD8^+^ T_EFF_ cells.

In a previous study, the research team led by Professor Hua Yu of the Beckman Research Institute and City of Hope Comprehensive Cancer Center (Duarte, CA, USA) demonstrated that STAT3 regulates FAO, which enhances breast cancer stem cell phenotype and promotes chemoresistance.^14^ Similar to cancer stem cells, CD8^+^ T_EFF_ cells can use exogenous fatty acids. Therefore, Professor Yu and colleagues believe that excessive fatty acids caused by obesity may also become the energy source of CD8^+^ T_EFF_ cells, which in turn affects the glycolysis of these cells. Additionally, since the inhibition of STAT3 can enhance Th1 antitumor immune response by inducing IFN‐γ production, they speculated that STAT3 may inhibit the activity of CD8^+^ T_EFF_ cells by converting their metabolism from glycolysis to FAO and thereby promoting the initiation and progression of obesity‐associated breast cancer.

In a study recently published in *Cell Metabolism*, entitled “STAT3 activation‐induced fatty acid oxidation in CD8^+^ T effector cells is critical for obesity‐promoted breast tumor growth”,^15^ Professor Yu and colleagues validated the above hypothesis. The mouse mammary tumor virus (MMTV)‐polyoma virus middle T antigen (PyMT) transgenic mice that could develop spontaneous mammary tumors were chosen for this study. The authors used a high‐fat diet to induce obesity in these mice. Compared with MMTV‐PyMT mice fed with a low‐fat diet, the high‐fat diet‐induced obese mice had earlier occurrence of breast cancer. Additionally, the weight of the breast tumors was much heavier and lung metastases were more likely to occur in the high‐fat diet‐induced obese mice. This was accompanied by a decrease in the absolute number of activated CD8^+^ T cells and the function of CD8^+^ T_EFF_ cells were inhibited (shown as a significant decrease in the percentage of CD8^+^ T cells expressing functional markers, including IFN‐γ, granzyme B, and CD107a) in the tumors developed in the high‐fat diet‐induced obese mice. These findings suggest that obesity may promote breast cancer initiation and progression by inhibiting breast cancer‐infiltrating CD8^+^ T_EFF_ cells. Next, the authors generated MMTV‐PyMT mice with either Stat3^+/+^ or Stat3^−/−^ to examine the role of STAT3 in T cells in the development of obesity‐associated breast cancer. They found that the tumor burden and incidence of lung metastasis caused by obesity in MMTV‐PyMT mice were significantly reduced when the STAT3 function in T cells was less. Furthermore, the authors utilized an in vivo STAT3 small interfering RNA (siRNA) delivery approach developed previously (linking STAT3 siRNA to the aptamer that selectively binds to cytotoxic T lymphocyte‐associated antigen 4 [CTLA4])^10^ to specifically inhibit STAT3 activity in T cells of MMTV‐PyMT mice. Similarly, compared with the control MMTV‐PyMT mice treated with CTLA4‐luciferase siRNA, CTLA4‐STAT3 siRNA treatment in high‐fat diet‐fed MMTV‐PyMT mice restricted the growth of breast cancer and promoted the tumor infiltration of functional CD8^+^ T_EFF_ cells. Since CD8^+^ T_EFF_ cells can use fatty acids as an energy source through FAO,^16^ the authors evaluated whether STAT3 could also upregulate FAO in CD8^+^ T_EFF_ cells to inhibit IFN‐γ production and its induced Th1 mediators. They analyzed the expression of Carnitine palmitoyltransferase 1B (CPT1B), which encodes the rate‐limiting enzyme of FAO and is a direct target gene of STAT3, in breast cancer infiltrating CD3^+^CD8^+^ T cells from MMTV‐PyMT mice with either Stat3^+/+^ or Stat3^−/−^. They found that when the function of STAT3 in infiltrating breast CD3^+^CD8^+^ T cells was lost, the expression level of CPT1B was decreased. In addition, ablating STAT3 in CD8^+^ T cells led to the upregulation of CD8^+^ T_EFF_ cell‐functional markers (IFNγ, granzyme B, and CD107a), which was also accompanied by an inhibition of FAO in the breast cancers from the MMTV‐PyMT mice. This evidence suggests that STAT3 mediates CD8^+^ T_EFF_ cell inhibition in breast cancer by promoting FAO. The preliminary study by Professor Yu showed that leptin could activate STAT3 in breast cancer stem cells and thereby increase FAO activity.^14^ In the present study, Profesor Yu and colleagues used antileptin antibodies to treat MMTV‐PyMT mice which were fed with a high‐fat diet. These results showed that tumor growth was restricted and the function of tumor CD8^+^ T_EFF_ cells was significantly enhanced. In other words, leptin neutralization in MMTV‐PyMT obese mice decreases STAT3 activity in CD8^+^ T_EFF_ cells, which leads to a decrease in FAO activity.

This study found that the increased FAO driven by leptin through STAT3 plays a critical role in inhibiting the glycolysis of CD8^+^ T_EFF_ cells thereby promoting obesity‐associated breast cancer initiation and progression. However, some limitations do exist in this study. First, the authors did not directly prove that increasing STAT3 could upregulate T cell FAO in the absence of other stimuli, although they used many different approaches to prove the importance of STAT3 in FAO upregulation. The data of the present study indicate that STAT3 could upregulate CPT1B, which could induce the upregulation of FAO in CD8^+^ T cells. However, the inhibition of CPT1B expression by CTLA4‐STAT3 siRNA only showed a weak inhibitory effect on tumor growth in vivo. In this special case, CPT1B was only silenced in some tumor‐infiltrated T cells, but almost all CD8^+^ T cells were activated. Therefore, in addition to FAO, there may be other approaches also involved in suppressing Th1 antitumor immune responses of CD8^+^ T cell. Second, the present study used T cells from db/db (Lepr‐KO) mice with immune abnormalities, so the potential complications caused by immune abnormalities during mouse development cannot be ruled out. We recommend using T cells from T cell conditional Lepr‐KO mice for related experiments. Despite these limitations, this study still provides a novel mechanical link between obesity and breast cancer via leptin and increased FAO in CD8^+^ T_EFF_ cells. These findings may lead to novel therapeutic strategies to reactivate antitumor CD8^+^ T cells in obesity‐associated breast cancer.

## Disclosure

The author declares no competing interests.

## References

[1] Hanahan D , Weinberg RA . Hallmarks of cancer: The next generation. Cell 2011; 144 (5): 646–74. 10.1016/j.cell.2011.02.013. 21376230

[2] Cheng C , Geng F , Cheng X , Guo D . Lipid metabolism reprogramming and its potential targets in cancer. Cancer Commun 2018; 38 (1): 27 10.1186/s40880-018-0301-4. PMC5993136 29784041

[3] Vinay DS , Ryan EP , Pawelec G *et al* Immune evasion in cancer: Mechanistic basis and therapeutic strategies. Semin Cancer Biol 2015; 35 (Suppl): S185–S98. 10.1016/j.semcancer.2015.03.004. 25818339

[4] Yan L , Zhang W . Precision medicine becomes reality‐tumor type‐agnostic therapy. Cancer Commun 2018; 38 (1): 6 10.1186/s40880-018-0274-3. PMC5953403 29764494

[5] Nakagawa H , Umemura A , Taniguchi K *et al* ER stress cooperates with hypernutrition to trigger TNF‐dependent spontaneous HCC development. Cancer Cell 2014; 26 (3): 331–43. 10.1016/j.ccr.2014.07.001. 25132496 PMC4165611

[6] Kuo CY , Ann DK . When fats commit crimes: Fatty acid metabolism, cancer stemness and therapeutic resistance. Cancer Commun 2018; 38 (1): 47 10.1186/s40880-018-0317-9. PMC6042406 29996946

[7] Wang Z , Aguilar EG , Luna JI *et al* Paradoxical effects of obesity on T cell function during tumor progression and PD‐1 checkpoint blockade. Nat Med 2019; 25 (1): 141–51. 10.1038/s41591-018-0221-5. 30420753 PMC6324991

[8] Rossi JF , Ceballos P , Lu ZY . Immune precision medicine for cancer: A novel insight based on the efficiency of immune effector cells. Cancer Commun 2019; 39 (1): 34 10.1186/s40880-019-0379-3. PMC6567551 31200766

[9] Herrmann A , Kortylewski M , Kujawski M *et al* Targeting Stat3 in the myeloid compartment drastically improves the in vivo antitumor functions of adoptively transferred T cells. Cancer Res 2010; 70 (19): 7455–64. 10.1158/0008-5472.CAN-10-0736. 20841481 PMC3058618

[10] Herrmann A , Priceman SJ , Swiderski P *et al* CTLA4 aptamer delivers STAT3 siRNA to tumor‐associated and malignant T cells. J Clin Invest 2015; 125 (6): 2547 10.1172/JCI82555. PMC4518694 26030229

[11] Yu H , Pardoll D , Jove R . STATs in cancer inflammation and immunity: A leading role for STAT3. Nat Rev Cancer 2009; 9 (11): 798–809. 10.1038/nrc2734. 19851315 PMC4856025

[12] Chang CH , Qiu J , O'Sullivan D *et al* Metabolic competition in the tumor microenvironment is a driver of cancer progression. Cell 2015; 162 (6): 1229–41. 10.1016/j.cell.2015.08.016. 26321679 PMC4864363

[13] Chowdhury PS , Chamoto K , Kumar A , Honjo T . PPAR‐induced fatty acid oxidation in T cells increases the number of tumor‐reactive CD8(+) T cells and facilitates anti‐PD‐1 therapy. Cancer Immunol Res 2018; 6 (11): 1375–87. 10.1158/2326-6066.CIR-18-0095. 30143538

[14] Wang T , Fahrmann JF , Lee H *et al* JAK/STAT3‐regulated fatty acid beta‐oxidation is critical for breast cancer stem cell self‐renewal and chemoresistance. Cell Metab 2018; 27 (1): 136–50 e5. 10.1016/j.cmet.2017.11.001. 29249690 PMC5777338

[15] Zhang C , Yue C , Herrmann A *et al* STAT3 activation‐induced fatty acid oxidation in CD8(+) T effector cells is critical for obesity‐promoted breast tumor growth. Cell Metab 2020; 31 (1): 148–61 e5. 10.1016/j.cmet.2019.10.013. 31761565 PMC6949402

[16] O'Sullivan D , van der Windt GJ , Huang SC *et al* Memory CD8(+) T cells use cell‐intrinsic lipolysis to support the metabolic programming necessary for development. Immunity 2014; 41 (1): 75–88. 10.1016/j.immuni.2014.06.005. 25001241 PMC4120664

